# A mini review of transforming dementia care in China with data-driven insights: overcoming diagnostic and time-delayed barriers

**DOI:** 10.3389/fnagi.2025.1554834

**Published:** 2025-03-03

**Authors:** Pinya Lu, Xiaolu Lin, Xiaofeng Liu, Mingfeng Chen, Caiyan Li, Hongqin Yang, Yuhua Wang, Xuemei Ding

**Affiliations:** ^1^Fujian Provincial Engineering Research Centre for Public Service Big Data Mining and Application, Fujian Provincial University Engineering Research Center for Big Data Analysis and Application, Fujian Normal University, Fuzhou, China; ^2^Department of Radiology, Shengli Clinical Medical College of Fujian Medical University, Fujian Provincial Hospital, Fuzhou University Affiliated Provincial Hospital, Fuzhou, China; ^3^Department of Neurology, Fujian Provincial Hospital, Fuzhou University Affiliated Provincial Hospital, Fuzhou, China; ^4^Key Laboratory of OptoElectronic Science and Technology for Medicine of Ministry of Education, Fujian Provincial Key Laboratory for Photonics Technology, Fujian Normal University, Fuzhou, China; ^5^Intelligent Systems Research Centre, School of Computing, Engineering and Intelligent Systems, Ulster University, Londonderry, United Kingdom

**Keywords:** dementia, Alzheimer’s disease, China dementia care pathway, computational strategy, machine learning, optimization, interpretability

## Abstract

**Introduction:**

Inadequate primary care infrastructure and training in China and misconceptions about aging lead to high mis−/under-diagnoses and serious time delays for dementia patients, imposing significant burdens on family members and medical carers.

**Main body:**

A flowchart integrating rural and urban areas of China dementia care pathway is proposed, especially spotting the obstacles of mis/under-diagnoses and time delays that can be alleviated by data-driven computational strategies. Artificial intelligence (AI) and machine learning models built on dementia data are succinctly reviewed in terms of the roadmap of dementia care from home, community to hospital settings. Challenges and corresponding recommendations to clinical transformation are then reported from the viewpoint of diverse dementia data integrity and accessibility, as well as models’ interpretability, reliability, and transparency.

**Discussion:**

Dementia cohort study along with developing a center-crossed dementia data platform in China should be strongly encouraged, also data should be publicly accessible where appropriate. Only be doing so can the challenges be overcome and can AI-enabled dementia research be enhanced, leading to an optimized pathway of dementia care in China. Future policy-guided cooperation between researchers and multi-stakeholders are urgently called for dementia 4E (early-screening, early-assessment, early-diagnosis, and early-intervention).

## 1 Introduction

Nowadays 16.99 million people in China are living with dementia [63–70% diagnosed with Alzheimer’s disease (AD)] (Wang et al., 2024). Without disease-modifying Alzheimer’s therapies, early diagnosis is critical to slowing disease progression and enhancing quality of life (Breijyeh and Karaman, 2020; Liss et al., 2021). In response, the National Health Commission of China, together with various medical organizations, has established a diagnostic process tailored to the Chinese population (Tian et al., 2019, 2021; National Center for Neurological Disorders et al., 2024) and is promoting the development of cognitive disorder treatment centers in township institutions (National Center for Neurological Disorders et al., 2024).

**Figure 1:**
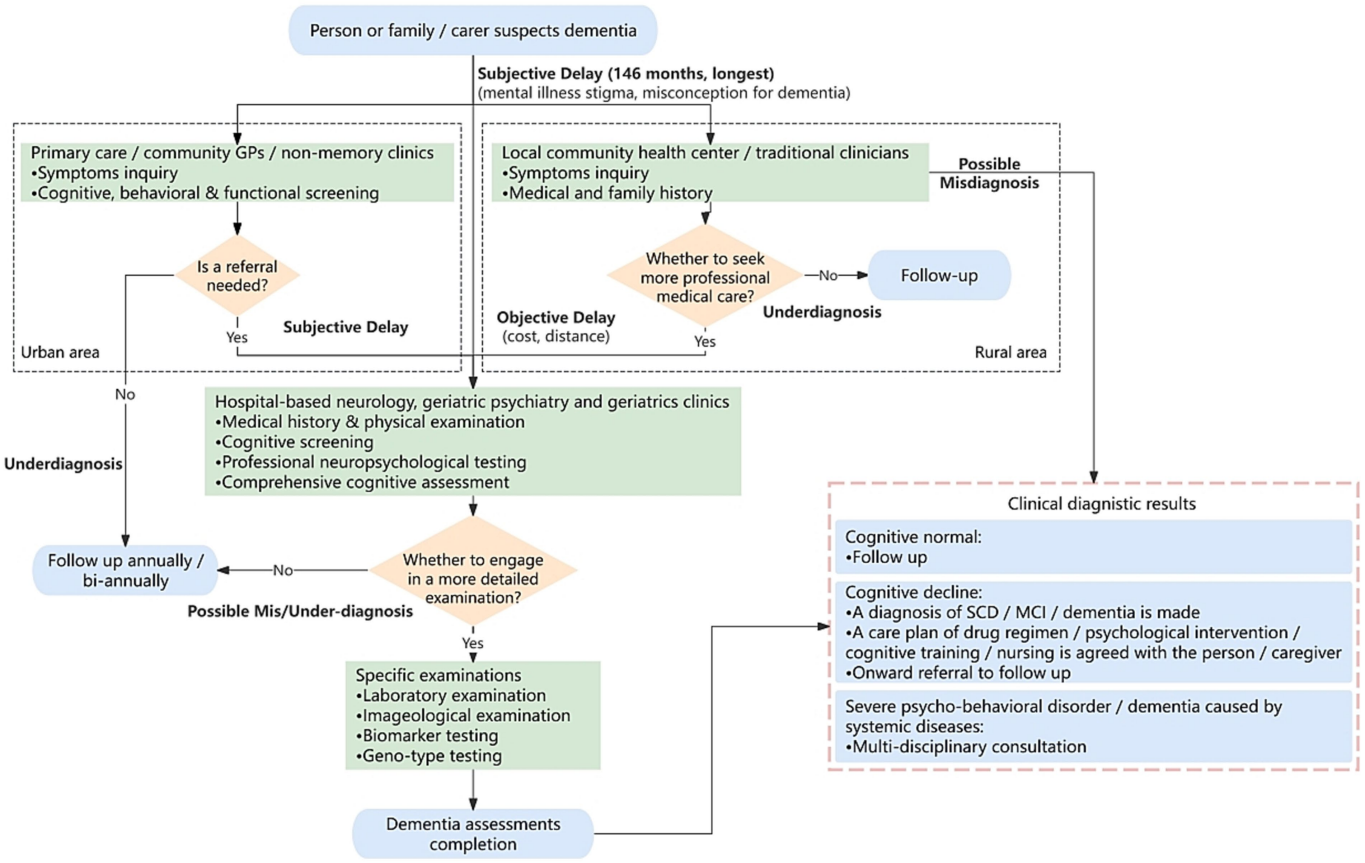
Flowchart of China dementia care pathway. Bolds: potential mis/under-diagnosis and time delay that could be alleviated by data-driven computational strategy. Flowchart proposed in the basis of studies in Tian et al. (2019, 2021), Zhang et al. (2020), Dementia and Cognitive Impairment Group of Chinese Society of Neurology, Cognitive Disorders Committee of Neurology Branch of Chinese Medical Doctor Association (2023), Huang et al. (2024), and National Center for Neurological Disorders et al. (2024). Blue rounded box: pathway starting/ending. Green box: dementia screening/assessing/examining. Orange diamond: if-then-else condition. GP, general practitioner; SCD, subjective cognitive decline; MCI, mild cognitive impairment.

However, many patients delay seeking medical care due to misconceptions about aging, dementia-related stigma, financial and/or distant issues, and inadequate primary healthcare, especially in rural areas of China, resulting in long waiting time of 24 to 146 months and significant cognitive decline (Zhao et al., 2016; Mattke et al., 2023; Olwage, 2024). Additionally, China lacks dementia-related specialists and hospital memory clinics (Wu and Lam, 2016). Only 24.7% general practitioners (GPs) received relevant training and about 60% reported feeling confident in providing diagnostic advice on dementia, leading to a high misdiagnosis rate (Li et al., 2021). Therefore, this study integrates expert consensus and prevention guidelines (Tian et al., 2019, 2021; Zhang et al., 2020; Huang et al., 2024; National Center for Neurological Disorders et al., 2024) into a comprehensive China dementia care pathway (Figure 1), highlighting two critical barriers: mis/under-diagnoses and time delays (bolds in Figure 1).

Specifically, for those patients with subjective cognitive decline (SCD) or mild cognitive impairment (MCI), in urban areas, the initial consultation usually occurs at a community primary hospital (indicated in the left black-dotted box in Figure 1). While in rural areas, patients may visit a local health center (if there is) or consult traditional Chinese medical practitioners (Quail et al., 2020) (see the right black-dotted box). If suspicions persist, they may be referred to secondary or specialized hospitals. Whereas some patients skip primary care and seek comprehensive evaluations at higher-level hospitals directly, including medical history, physical exams, neuropsychological assessments, blood tests, computed tomography or magnetic resonance imaging (MRI) scans, positron emission tomography (PET) scans or cerebrospinal fluid (CSF) analysis. With the completion of all tests, the diagnosis and corresponding care plan will be made (bottom-right pink-dotted box).

In this pathway, both objective and subjective factors contribute to mis/under-diagnoses and time delays. Advancements in computing science, particularly data-driven methods, can help reduce subjective errors while improving diagnostic accuracy and efficiency (Muhammad et al., 2021; Rodrigues et al., 2023). Therefore, these approaches hold promise in addressing the existing challenges in China dementia care pathway (Devenney et al., 2024).

## 2 From home to hospital: digital health care

### 2.1 Scenarios in home and community

Digital neuropsychological testing can reduce errors from manual record-keeping and evaluator biases, improving diagnostic precision and streamlining the process, while also enabling better data storage and remote management (Dementia and Cognitive Impairment Group of Chinese Society of Neurology, Cognitive Disorders Committee of Neurology Branch of Chinese Medical Doctor Association, 2023). Some existing digital strategies are the computer assessment of MCI (CAMCI-Research, 2016), Cambridge neuropsychological test automated battery paired associates learning (CANTAB Assessments, 2012), and CogState digital cognitive assessment (CogState, 2013) etc., where CogState brief battery is a highly automated and standardized tool, lasting only 15 min and assessing multiple cognitive functions (Staffaroni et al., 2020). These tools support less experienced GPs in primary care, reducing misdiagnosis rates.

Each neuropsychological assessment focuses on specific areas. Previous research showed that some assessments performed poorly in certain cognitive domains, whereas multiple assessments together cover a broader range (Yin et al., 2015). However, comprehensive neuropsychological assessment is time-consuming, prompting computational studies into the most effective assessment combinations for diagnosing MCI and AD (Hemmy et al., 2020). For instance, Bucholc et al. (2019) used support vector machine (SVM) to assess dementia severity, achieving 83% accuracy by combining mini-mental state examination (MMSE), Montreal cognitive assessment (MoCA), functional activities questionnaire (FAQ), and AD assessment scale. Similarly, McCombe et al. (2022) employed random forest using nine selected sub-items from FAQ, MMSE, and the global deterioration scale, achieving an area under the receiver operating characteristic curve (AUC) of 0.865 for classifying cognitive normal (CN), MCI, and AD. These methods improve diagnostic accuracy even using fewer scales, demonstrating the potential of data-driven computational approaches to assist GPs in reducing misdiagnoses and shortening clinical management times.

Moreover, emerging data-driven techniques have shown great potential in the early detection, caregiver support and personalized management of AD, with the possibility of being widely adopted in home and community scenarios. For instance, wearable devices, as a non-invasive technology, collect real-time physical data from patients, e.g., retinal imaging (Wang et al., 2021; Gaire et al., 2024), language (Lindsay et al., 2021), hearing (Bucholc et al., 2021), and gait (Duan et al., 2023) etc. These devices, combined with artificial intelligence (AI) techniques, analyze patient’s physical condition, lighten care burdens, and aid doctors in making well-informed decisions remotely (Qi et al., 2022; Salehi et al., 2022; Vrahatis et al., 2023; Borna et al., 2024; Chen et al., 2024). A study used gait data collected via seven wearable devices served for 77 CN and 68 MCI subjects, with advanced machine learning (ML) models achieving classification accuracy of 0.73 in dual-task walking and 0.66 in normal walking (Jeon et al., 2023). In addition, other smart technologies can assist clinicians and patients through interactive devices and distributed systems, such as leveraging sensors in homes or care facilities to collect patient and environmental data and providing feedback to patients. For instance, Munteanu et al. (2022) designed an intelligent assistance system that uses AI to recognize human activities in videos. This system can detect when AD patients eat or drink and remind them with a voice message if they forget or overconsume. It also enables caregivers to remotely supervise and manage the patient’s nutrition plan. Integrating these technologies into routine dementia management can improve care efficiency and enhance quality of patients’ life (Gillani and Arslan, 2021). Therefore, data-driven computational approaches are efficient for early dementia detection and personalized care, optimizing the dementia care pathway.

### 2.2 Scenario in hospital setting

Patients with referrals can further undergo comprehensive examinations at a hospital to determine the dementia type and corresponding treatment planning. Specifically, neuroimaging examinations can identify changes in the brain. Currently, structural MRI (sMRI) is the most important imaging detection method of prodromal AD (pAD), as it provides valuable imaging markers (Dementia and Cognitive Impairment Group of Chinese Society of Neurology, Cognitive Disorders Committee of Neurology Branch of Chinese Medical Doctor Association, 2023). A systematic review indicates that, for identifying MCI, the total hippocampal volume contributes a sensitivity of 0.73 and specificity of 0.71; medial temporal lobe atrophy provides a sensitivity of 0.64 and specificity of 0.65; and lateral ventricular volume presents a sensitivity of 0.57 and specificity of 0.60 (Lombardi et al., 2020). Nowadays, majority of imaging analyses utilize various deep learning (DL) methods to capture brain structural and pathological changes in sMRI images, eventually improving the accuracy of dementia diagnosis and prognosis (Yamanakkanavar et al., 2020; Frizzell et al., 2022; Borchert et al., 2023; Xu et al., 2023). Crucially, these decision-making support techniques significantly reduce errors caused by clinical fatigue or negligence in practice (Zhou et al., 2024).

Unlike MRI, a PET scan is more expensive and invasive, but it reveals molecular metabolic activity and brain function, making it a valuable tool for diagnosing and assessing neurological disorders like dementia (Borchert et al., 2023; Ricci et al., 2020; Bao et al., 2021). It turns out that conventional ML and DL has been effectively utilized for PET image analytics to detect lesion size, morphology, and changes over time and further to improve the accuracy of early-stage dementia diagnosis and prognosis (Tang et al., 2024). For instance, SVM achieved >85% accuracy in detecting AD-specific hypometabolic patterns with fluorodeoxyglucose (FDG)-PET and outperforming sMRI (Ferreira et al., 2017; De Carli et al., 2019). It also performed promising in distinguishing AD vs. CN (>86% accuracy) and MCI vs. CN (>78.8% accuracy) as well as predicting MCI-to-AD conversions within 12–60 months (72–80% accuracy), all based on FDG-PET images (Pan et al., 2019; Li et al., 2019; Shen et al., 2019; Teng et al., 2020). Similarly, applying SVM to amyloid-PET data achieved >85% accuracy for predicting MCI-to-AD conversions and diagnosing AD (Nozadi and Kadoury, 2018; Yang et al., 2020). In terms of DL, convolutional neural networks have presented a range of accuracy of 75–100% on FDG-and amyloid-PET images, depending on the variables of data size, imaging modality, and image preprocessing (Ding et al., 2019; Huang et al., 2019; Son et al., 2020). These findings highlight the potential of ML to enhance PET imaging analytics for AD diagnosis and prognosis (Borchert et al., 2023) in hospital setting. In addition, leveraging the combination of PET and MRI images enables more comprehensive assessments. More studies regarding DL on PET/MRI focus on image segmentation and reconstruction, pathological features visualization, thereby facilitating dementia detection and prediction (Dyrba et al., 2021; Zhao et al., 2023; Zhou et al., 2024).

Another rich source of biomarkers for AD comes from CSF because it reflects changes in the central nervous system caused by neuronal metabolic disturbances (McGrowder et al., 2021; Bouwman et al., 2022; Ardanaz et al., 2022). CSF Aβ protein concentration has shown strong diagnostic performance, with sensitivity and specificity of 0.86 and 0.80 for distinguishing AD from CN, and 0.79 and 0.61 for differentiating AD from non-AD dementias. Other CSF biomarkers, such as T-tau and P-tau181, have also demonstrated high diagnostic efficacy (Tian et al., 2019). Recent studies have pointed out that the clinically diagnosed AD group may include patients with other types of dementia, while the control group often contains individuals with other neurological disorders, complicating the definition of cut-off values between the groups (Bellomo et al., 2021). To address this, Bellomo et al. used unsupervised ML methods to calculate unbiased cut-off values based on CSF biomarker distribution, reducing inter-laboratory variability and improving biochemical phenotyping. For instance, a study utilizing proximity extension-based assays to analyze CSF from dementia patients identified dopamine decarboxylase (DDC) as the most significantly dysregulated protein. DDC effectively classified Lewy body dementia (LBD) vs. CN with an AUC of 0.91, and LBD vs. AD with an AUC of 0.81. The study further established a biomarker panel comprising seven CSF proteins via the constructed classification model, achieving an improved AUC performance of 0.93 for differentiating LBD vs. AD (Del Campo et al., 2023). Subsequently, to determine the optimal combination of CSF biomarkers for predicting disease progression in AD and other neurodegeneration, a study analyzed data from 1,983 participants across three cohorts. Statistical analysis revealed that P-tau/Aβ42 is sufficient for predicting progression in AD with AUC performance greater than 0.87 (Salvadó et al., 2023). As such, data-driven computational strategies have made significant contributions in precise patient stratification, the discovery of novel biomarkers, and the identification of effective marker combinations that enhance clinical diagnosis, thereby further reducing dementia misdiagnosis rates.

Researchers are also exploring peripheral biomarkers, such as blood tests, which offer a less invasive and more accessible method for detecting pAD, with high sensitivity and specificity (Dementia and Cognitive Impairment Group of Chinese Society of Neurology, Cognitive Disorders Committee of Neurology Branch of Chinese Medical Doctor Association, 2023). The measurement of plasma Aβ protein concentrations demonstrates a combined sensitivity and specificity of 0.88 and 0.90, respectively, in distinguishing AD and MCI from CN. Similarly, plasma tau concentrations show a combined sensitivity and specificity of 0.96 and 0.93, respectively, for the same differentiation. These findings highlight plasma biomarkers as a promising option for AD diagnosis (Tian et al., 2021). A recent study using a Markov model to predict the dementia care burden in China from 2024 to 2043 indicated that using blood tests could significantly shorten dementia care pathway, typically in reducing patient waiting time for diagnosis (Mattke et al., 2023). Importantly, AI techniques can be utilized in analyzing high-dimensional and complex blood data (Lee and Lee, 2020; Palmqvist et al., 2024). For instance, an exciting research based on ML had identified a small set of blood transcripts capable of effectively distinguishing CN from those with neurodegenerative diseases, including AD (Huseby et al., 2022).

In terms of genetic biomarkers, it has already been reported that apolipoprotein E (ApoE) ε4 allele is associated with higher AD risk than the more common ApoE ε3 allele (Neu et al., 2017; Narasimhan et al., 2024), highlighting the importance of genetic testing in dementia assessment (Koriath et al., 2021). However, challenges remain in translating human genetic findings [such as genome-wide association studies (GWAS)] into the pathobiology and therapeutic discoveries for AD. To address this, a DL framework was proposed to identify disease-associated genes (Xu et al., 2022). This framework identified 156 AD-related genes, enriched in druggable molecules, and discovered four drugs linked to reduced AD incidence. These breakthroughs emphasize the potential of DL in both understanding AD pathobiology and in identifying genetic markers for early disease prediction and prevention. Given AD’s polygenic nature, polygenic risk scores (PRS) assess genetic susceptibility. Zhou et al. (2023) used DL to model genetic data more comprehensively, showing clear advantages over traditional PRS and least absolute shrinkage and selection operator models in identifying genetic risk and uncovering biological mechanisms. In addition, the integration of computational models into genetic evaluation enables the early identification of individuals at higher risk for AD, allowing for targeted prevention strategies and more timely interventions. In summary, computational models excel at processing large-scale datasets from genomics and proteomics, not only enabling the discovery of novel biomarkers but also predicting their associations with disease onset and progression. By detecting complex data patterns, these models may uncover insights overlooked by human experts, thereby enhancing screening efficiency for dementia patients and high-risk populations and consequently speeding dementia care pathway (Javaid et al., 2022).

In clinical settings, single-modal data typically provides only a partial view of the disease and cannot comprehensively reflect its full scope (Elazab et al., 2024). Multi-modal data and corresponding techniques can capture various aspects of the disease and biomarkers related to AD pathology, leading to more accurate and personalized diagnostic results (Muhammad et al., 2021). Recently, a considerable AI-model built on multimodal data over diverse cohorts has been proposed to differentiate dementia etiologies (Xue et al., 2024), achieving a microaveraged AUC of 0.94 for classifying CN, MCI, and dementia. In a random subset of 100 cases, the AUC of neurologist assessments increased by 26.25% with AI assistance compared to assessments made by neurologists alone, underscoring the significant enhancement that AI provides in supporting expert evaluations for dementia diagnosis. In addition, Rahim et al. (2024) used multimodal data to predict patient outcomes 3 years later with a DL model, achieving 88% generalization accuracy and an AUC of 0.88. This kind of multimodal data study of AD progression can provide information support for clinicians to intervene in treatment. Hospitals in China are actively engaging in multi-sectional cohort studies to collect, consolidate and integrate multimodal data.

Additionally, some studies on AD care recommendations use additional care assessments, such as those related to daily living care problems, behavioral and psychological symptoms, and safety risks. For example, a study using a knowledge graph to develop a care recommendation system achieved 98.92% accuracy, providing decision support for personalized AD patient care and improving the care process (Sun et al., 2024).

## 3 Challenges and recommendations

### 3.1 Dilemma in clinical data

The complexity of dementia pathology challenges the acquirement of large-scale, clean data across multi-centers, thus hindering the translation of computational strategies to clinical settings (Afzal et al., 2019; Bron et al., 2022). Consequently, most studies rely on openly-sourced datasets like Alzheimer’s Disease Neuroimaging Initiative (Mueller et al., 2005), Australian Imaging Biomarkers & Lifestyle (Ellis et al., 2009), National Alzheimer’s Coordinating Center (Beekly et al., 2007), and UK BioBank (Ollier et al., 2005). Although ClinicalTrials.gov reports Chinese dementia cohort recruitment, patient data are still inaccessible.

Implementing data-driven strategies in China requires government-supported collaboration between medical and research institutions to build a multi-centered big-data platform integrating massive clinical records (National Center for Neurological Disorders et al., 2024). Poor data interoperability often brings redundant testing after referrals. Therefore, standardized data structuring and quality evaluation processes are essential to ensure data integrity and usability (Fan and Fu, 2023), enabling computational models to address clinical challenges like mis/under-diagnoses, drug efficacy, and health economics, ultimately providing more accurate and timely diagnoses for dementia patients (Xian et al., 2023; Gao et al., 2021).

### 3.2 Computational model reliability

Many computational models emphasize improving accuracy to support clinicians in making diagnostic decision, but the “black-box” issue (lack of interpretability) hinders trust from patients and doctors (Bron et al., 2022; Mirzaei and Adeli, 2022; Wang et al., 2023; Chen and Cheng, 2024). Other challenges include terminology inconsistencies, preprocessing methods variety, unclear evaluation criteria, and model optimization complexity (Bron et al., 2022). As a result, varying implementation details across studies hinder practical validation.

Recommendations include traditional ML algorithms (e.g., Bayesian networking, distance-based modeling), offering inherent interpretability but require extensive manual feature engineering (Ding et al., 2018; Yang et al., 2024). In contrast, post-hoc interpretable methods like class activation mapping (CAM) and Grad-CAM enhance transparency for complex models such as neural networks (Zhou et al., 2016; Selvaraju et al., 2017). Moreover, occlusion methods improve trust in AI by identifying key features through output image comparison (Kwak et al., 2022a,b). Crucially, before large-scale deployment of data-driven models, clinical requirements elicitation, policy guidance, and extensive validation are the foundation to ensure clinical relevance, reliability, and transparency.

## 4 Discussion

Clear evidence exists for using data-driven computational strategy to speed up clinical administration time and reduce mis/under-diagnosis rate, i.e., optimize China dementia care pathway. Therefore, dementia cohort study along with developing a center-crossed dementia platform in China should be strongly encouraged. Data should also be publicly accessible where appropriate. Only be doing so can the challenges be overcome and can AI-enabled dementia research be enhanced, thereby optimizing China dementia care pathway. Clinical transformation urgently requests substantive cooperation of multi-stakeholders, including computational researchers, medical professionals, healthcare specialists, policymakers, and industrial developers etc., across regions.

## Glossary

AD: Alzheimer’s disease
AI: Artificial intelligence
ApoE: Apolipoprotein E
AUC: Area under the receiver operating characteristic curve
CAM: Class activation mapping
CN: Cognitive normal
CSF: Cerebrospinal fluid
DDC: Dopamine decarboxylase
DL: Deep learning
FAQ: Functional activities questionnaire
FDG-PET: Fluorodeoxyglucose positron emission tomography
GP: General practitioner
LBD: Lewy body dementia
MCI: Mild cognitive impairment
ML: Machine learning
MMSE: Mini-mental state examination
MoCA: Montreal cognitive assessment
MRI: Magnetic resonance imaging
NCND: National center for neurological disorders
pAD: Prodromal AD
PRS: Polygenic risk scores
SCD: Subjective cognitive decline
sMRI: Structural magnetic resonance imaging
SVM: Support vector machine
